# Diffusion tensor imaging and tractography of the sciatic and femoral nerves in healthy volunteers at 3T

**DOI:** 10.1186/s13018-017-0690-0

**Published:** 2017-11-29

**Authors:** Yasushi Wako, Junichi Nakamura, Yawara Eguchi, Shigeo Hagiwara, Michiaki Miura, Yuya Kawarai, Masahiko Sugano, Kento Nawata, Kensuke Yoshino, Yasunari Toguchi, Yoshitada Masuda, Koji Matsumoto, Takane Suzuki, Sumihisa Orita, Seiji Ohtori

**Affiliations:** 1 Department of Orthopedic Surgery, Graduate School of Medicine, Chiba University, 1-8-1 Inohana, Chuo-ku, Chiba city, Chiba 260-8677 Japan; 2 Department of Orthopedic Surgery, National Hospital Organization Shimoshizu National Hospital, 934-5, Shikawatashi, Yotsukaido city, Chiba 284-0003 Japan; 3 Department of Radiology, Chiba University Hospital, 1-8-1 Inohana, Chuo-ku, Chiba city, Chiba 260-8677 Japan; 4 Department of Bioenvironmental Medicine, Graduate School of Medicine, Chiba University, 1-8-1 Inohana, Chuo-ku, Chiba city, Chiba 260-8677 Japan

**Keywords:** Diffusion tensor imaging, Tractography, Magnetic resonance imaging, Sciatic nerve, Femoral nerves, Hip joint, Healthy volunteers

## Abstract

**Background:**

The aim was to clarify the normal fractional anisotropy (FA) and apparent diffusion coefficient (ADC) values of the sciatic and femoral nerves at the level of the hip joint and to visualize the neural tracts with diffusion tensor imaging (DTI).

**Methods:**

Twenty-four healthy volunteers (12 men and 12 women, age 20–29 years) underwent DTI for visualization with tractography and quantification of FA and ADC values on a 3 Tesla MRI (*b* value = 800 s/mm^2^, motion probing gradient, 11 directions, time to repeat/echo time = 9000/72.6 ms, axial slice orientation, slice thickness = 3.0 mm with no inter-slice gap, field of view = 320 × 320 mm, 96 × 192 matrix, 75 slices, number of acquisitions = 4). Regions of interest in the sciatic nerve were defined at the femoral head, the S1 root, and the midpoint levels. The femoral nerve was evaluated at 3–4 cm proximal to the femoral head level.

**Results:**

The tractography of the sciatic and femoral nerves were visualized in all participants. The mean FA values of the sciatic nerve were increased distally from the S1 root level, through the midpoint, and to the femoral head level (0.314, 0.446, 0.567, *p* = 0.001, respectively). The mean FA values of the femoral nerve were 0.565. The mean ADC values of the sciatic nerves were significantly lower in the S1 root level than in the midpoint and the femoral head level (1.481, 1.602, 1.591 × 10^−3^ × 10^−3^ mm^2^/s, *p* = 0.001, respectively). The ADC values of the femoral nerve were 1.439 × 10^−3^ mm^2^/s. FA and ADC values showed moderate to substantial inter- and intra-observer reliability without significant differences in gender or laterality.

**Conclusion:**

Visualization and quantification of the sciatic and femoral nerves simultaneously around the hip joint were achieved in healthy young volunteers with DTI. Clinical application of DTI is expected to contribute to hip pain research.

## Background

Diffusion tensor imaging (DTI) is an advanced magnetic resonance (MR) imaging technique, which provides information about the microstructure of nerve fibers noninvasively [1]. With the application of the appropriate magnetic field gradients, MR imaging can be sensitized to the thermally driven random motion of water molecules in the direction of the field gradient. This technique is called diffusion-weighted imaging (DWI) [2]. Many materials have intrinsic structural properties that hinder diffusion so that the diffusivity of the water molecules is greater in some directions than in others. This property is known as anisotropy. If there is no directional variation in the diffusion rate, diffusion is said to be isotropic. Biologic tissues often are anisotropic because structures such as cell membranes restrict the motion of water molecules. DWI typically shows diffusion information in only one direction. However, DTI can be used to quantify anisotropy in an anisotropic sample [2]. For this quantification, fractional anisotropy (FA) is the parameter used most frequently. The FA value of a completely isotropic sample is 0. As the anisotropy increases, the FA value increases, and a completely anisotropic sample has a value equal to 1. The apparent diffusion coefficient (ADC) value is obtained by quantifying the intensity of the overall diffusion, although anisotropy cannot be assessed due to the lack of directionality information in the calculation.

DTI can visualize neural tracts with diffusion tensor tractography, which has been used mainly for imaging of the central nervous system to visualize white matter tracts in neuronal disorders such as stroke, multiple sclerosis, and/or chronification of migraine [3, 4]. DTI has also been applied to peripheral nerves. In 2004, Skorpil et al. [5] first reported visualization of the sciatic nerves in three healthy volunteers with DTI on a 1.5-Tesla MR scanner. After that, several researchers succeeded in visualization and quantification of various peripheral nerves such as the median, ulnar, peroneal, tibial, and sural nerves [6–9]. Eguchi and Oikawa et al. performed visualization of the lumbar nerve roots, reporting a decrease in FA values and an increase in ADC values at the compression site [10–14]. Currently, nerve conduction studies prevail for the evaluation of peripheral nerves, although it is limited to distal peripheral nerves, relying on target innervation. Nonetheless, clinical relevance of DTI is expected to develop a non-invasive diagnostic tool for peripheral nerves.

The mechanism for hip pain has been unclear because of a lack of experimental studies. X-ray imaging is a standard method for diagnosing the advanced hip disorders. MR imaging is also useful for the early stage of degenerative articular lesion. However, there are no qualitative or quantitative imaging techniques for hip pain. Therefore, the authors have noticed DTI to build a foundation of hip pain research. The sciatic and femoral nerves are two major nerves of the lower limbs and may contribute to pain experienced from hip diseases such as piriformis muscle syndrome [15], osteoarthritis of the hip [16, 17], or osteonecrosis of the femoral head [18, 19]. However, the normal FA values of the sciatic and femoral nerves in young and healthy humans are not well studied. Furthermore, the sciatic and femoral nerves have not been visualized around the hip joint simultaneously with DTI.

The purposes of this study were to clarify the normal FA and ADC values for the sciatic and femoral nerves at the level of the hip joint and to visualize the neural tracts using DTI tractography.

## Methods

The research protocol was in compliance with the Helsinki Declaration, approved by the Research Ethics Committees of Graduate School of Medicine, Chiba University (reference number 657), and registered with the University Hospital Medical Information Network. Written informed consent was obtained from all study participants.

### Subjects

From June 2014 to February 2016, 24 healthy volunteers (12 men and 12 women) were enrolled. Inclusion criteria were age from 20 to 29 years and no history of medication, surgery, or lower limb symptoms such as numbness or pain. The characteristics of the healthy volunteers are shown in Table 1. Male participants were significantly younger, taller, and heavier than female participants.

**Table 1:** Characteristics of the healthy volunteers

Variable	Overall (*n* = 24)	Male (*n* = 12)	Female (*n* = 12)	*p* value
Age (years)	24.8 ± 2.6	25.9 ± 2.4	23.7 ± 2.4	0.03*
Height (cm)	163 ± 8	169 ± 4	157 ± 6	0.01*
Weight (kg)	55.5 ± 9.1	61.2 ± 7.2	49.8 ± 7.0	0.01*
BMI (kg/m^2^)	20.7 ± 2.5	21.3 ± 2.7	20.1 ± 2.2	0.37

Mean ± standard deviation. *BMI* body mass index

*Mann–Whitney’s *U* test

### MRI protocol

Magnetic resonance imaging was performed on a 3 Tesla MR imaging scanner (Discovery MR750; GE Healthcare, Milwaukee, Wisconsin). Participants were scanned in a supine position using a Sense XL Torso coil. Limbs were placed for a neutral position of the hip joints. DTI was performed using a special sensitivity array encoding protocol, with the factor = 2, chemical shift-selective suppression, and an echo-planar imaging sequence with a free-breathing scan technique. The image without diffusion encoding (*b* value = 0 s/mm^2^) was acquired to register diffusion-weighted volume when analyzing. The following imaging parameters were set: *b* value = 800 s/mm^2^, motion probing gradient (MPG), 11 directions, time to repetition (TR)/echo time (TE) = 9000/72.6 ms, flip angle = 90°, axial slice orientation, slice thickness = 3.0 mm with no inter-slice gap, field of view = 320 × 320 mm, 96 × 192 matrix, 75 slices, number of acquisitions = 4, and a 7-min 21-s scan time. For anatomic reference, a three-dimensional Multiple Echo Recombined Gradient Echo (MERGE) sequence was obtained with TR/TE = 30/12.5 ms, flip angle = 8°, field of view = 320 × 256 mm, imaging matrix = 320 × 288, number of excitations = 1, acquisition time of 8 min 11 s. With a bandwidth of ± 83.3 kHz, the seven TEs were obtained at 12.5–28.5 ms.

### Image analysis

**Fig. 1:**
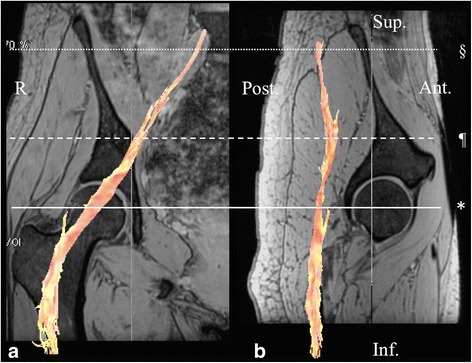
A fusion image of diffusion tensor tractography of the sciatic nerve with multiple echo recombined gradient echo imaging. **a** A coronal section of the femoral head in the right hip showing the sciatic nerve fibers from the S1 to the proximal femur in orange. **b** A sagittal section of the femoral head in the right hip showing the sciatic nerve fibers. The sciatic nerve was defined at the femoral head level (*), the S1 root level (§), and the midpoint level (¶). R right, Sup superior, Inf inferior, Ant anterior, Post posterior

**Fig. 2:**
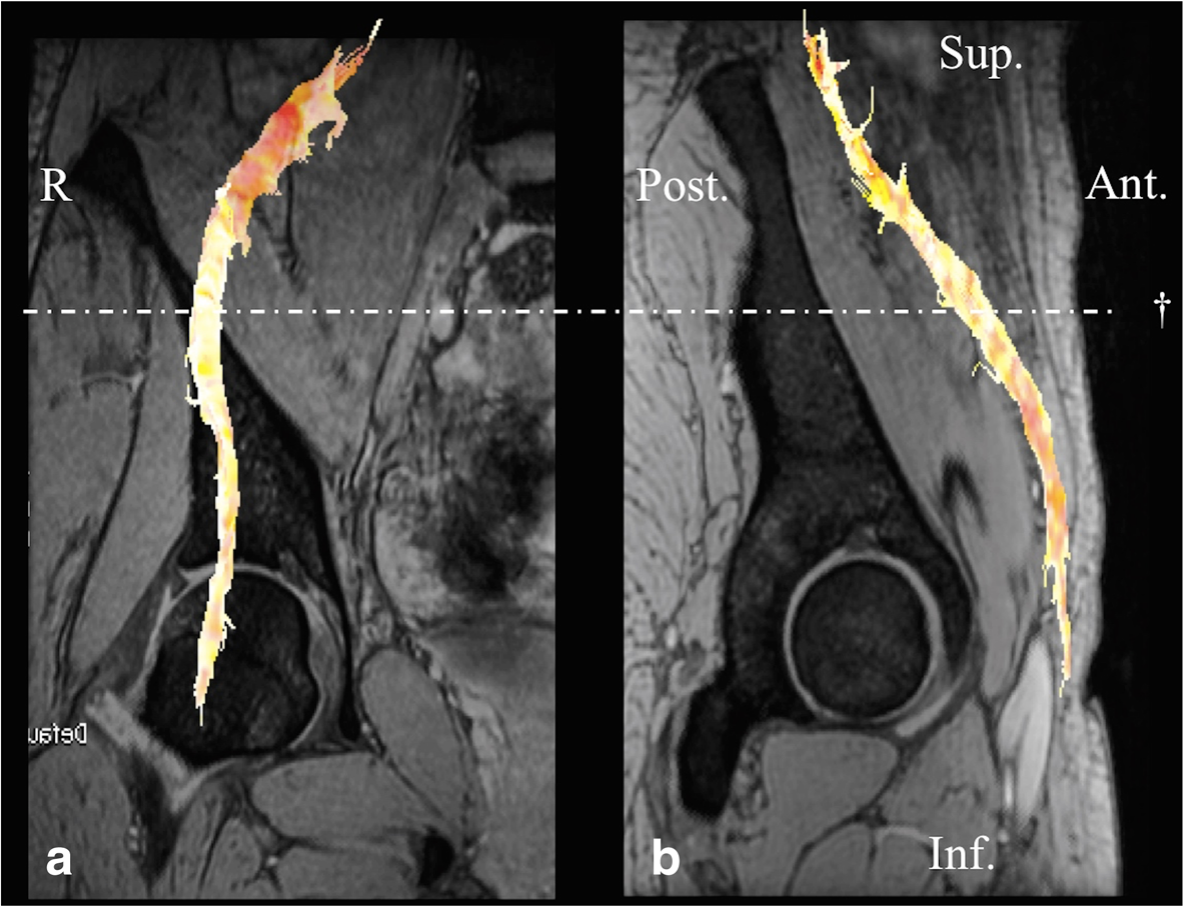
A fusion image of diffusion tensor tractography of the femoral nerve with multiple echo recombined gradient echo imaging. **a** A coronal section of the femoral head in the right hip showing the femoral nerve fibers in orange. **b** A sagittal section of the femoral head in the right hip showing the femoral nerve fibers. The femoral nerve was defined at 3–4 cm proximal from the femoral head level (†). R right, Sup superior, Inf inferior, Ant anterior, Post posterior

**Fig. 3:**
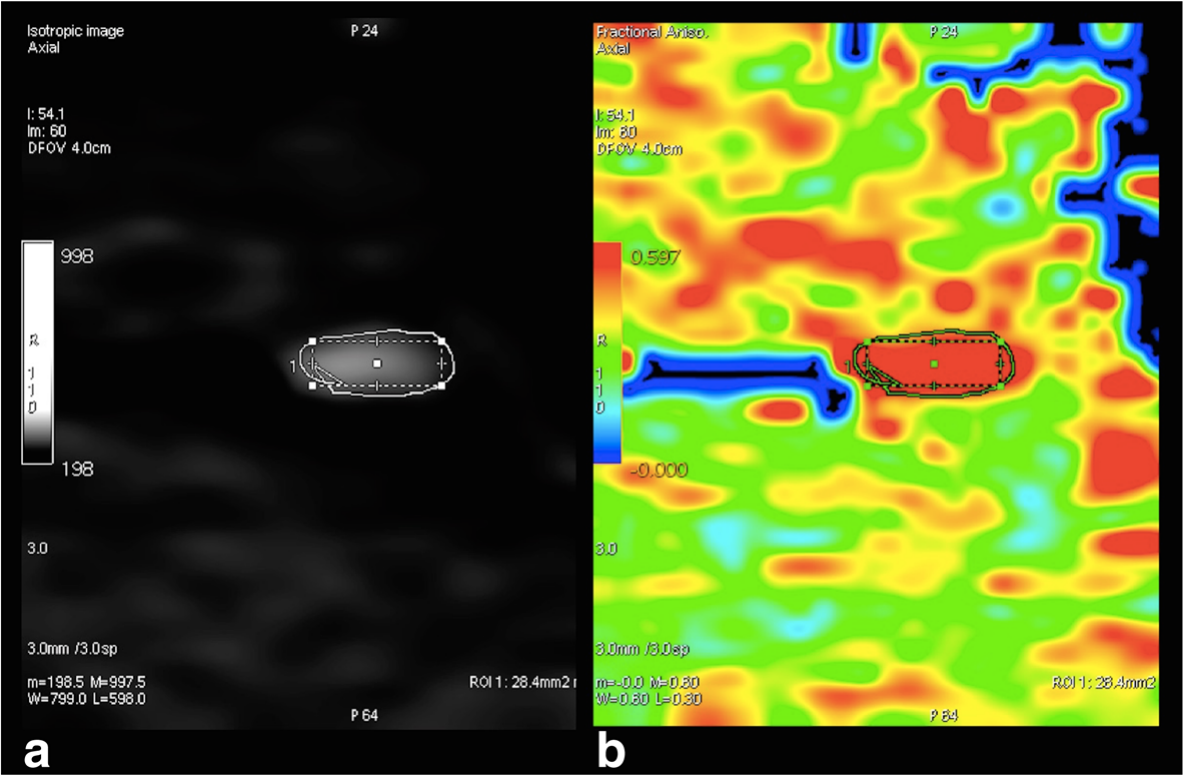
Region of interest placement. **a** An axial section of an isotropic image shows a high signal area, where the region of interest is placed in the nerve. **b** An axial section of the fractional anisotropy map shows a red area, indicating the nerve. **a** and **b** are in the same slices

Analysis was performed on a work station (Readyview, GE Healthcare, Milwaukee, Wisconsin) for tractography and FA and ADC mapping. The following parameters were used for fiber tracking: FA threshold, 0.2 and angle threshold 30°. DTI were first corrected for the eddy currents by registering them to the reference image (*b* = 0; no diffusion weighting) using the affine registration. The FA and ADC values were obtained using the following procedure: Initially, the sciatic nerve and the dorsal root ganglion of the S1 root were identified in an isotropic image in the coronal and sagittal planes (Fig. 1). Regions of interest (ROIs) of the sciatic nerve were defined at the femoral head, the S1 root, and the midpoint levels. Secondly, the ROI of the femoral nerve was defined at 3–4 cm proximal from the femoral head level because visualization of the nerve on this point was the clearest in all subjects (Fig. 2). The ROI was placed manually, and then, the FA and ADC values were measured automatically (Fig. 3). To minimize variation, the ROI was measured three times for each point and the average value was calculated. We measured the FA and ADC values on both sides of the body for laterality calculations. We also examined gender differences.

### Statistical analyses

Inter-observer reliabilities were measured by two observers. Intra-observer reliabilities were determined by measuring FA and ADC values in a similar fashion 2 weeks later. Inter- and intra-observer reliabilities were evaluated by intra-class correlation coefficients (ICCs). The following ICC categories were used for interpretation: 0.00–0.10 = virtually none, 0.11–0.40 = slight, 0.41–0.60 = fair, 0.61–0.80 = moderate, and 0.81–1.00 = substantial agreement. Differences in laterality and gender were compared by Mann–Whitney’s *U* test, and a *p* value of less than 0.05 was considered statistically significant (BellCurve for Excel, Social Survey Research Information Co., Ltd.). The change in the FA and ADC values with respect to age were calculated by a simple regression analysis.

## Results

The tractography of the sciatic and femoral nerves were visualized in all the 24 participants. DTI tractography fusing with the MERGE image was shown in Figs. 1 and 2.

For inter-observer reliability, the mean ICCs of the FA values were 0.889 at the S1 root, 0.722 at the midpoint and 0.701 at the femoral head of the sciatic nerve, and 0.729 in the femoral nerve. The mean ICCs of the ADC values at the four points were 0.767, 0.680, 0.695, and 0.806, respectively. For intra-observer reliability, the mean ICCs of the FA values at the four points were 0.930, 0.842, 0.795, and 0.829, respectively. The mean ICCs of the ADC values at the four points were 0.834, 0.787, 0.812, and 0.829, respectively.

**Fig. 4:**
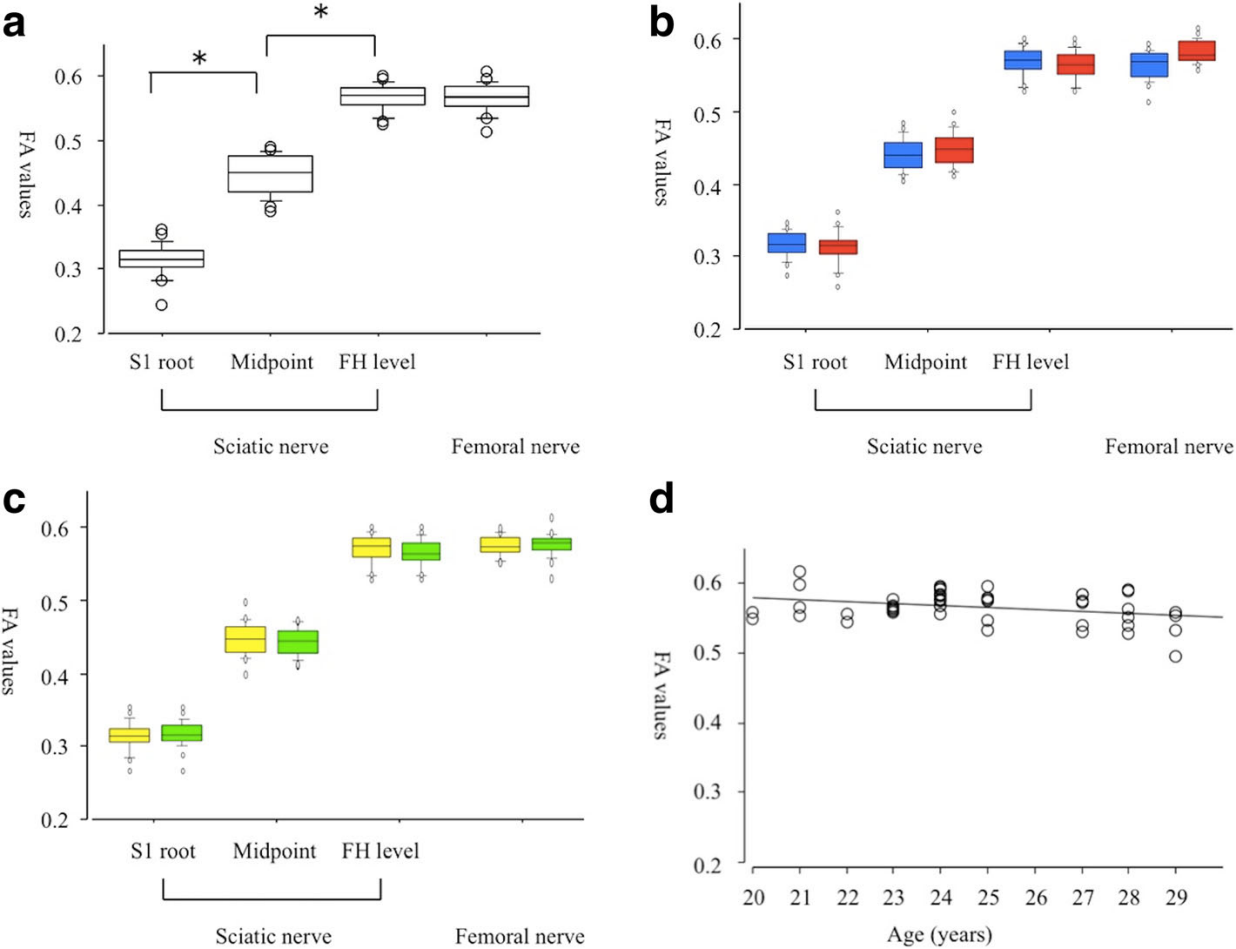
The fractional anisotropy values from the sciatic and femoral nerves. **a** The overall distribution of FA values. The FA value of the sciatic nerve was decreased significantly from the femoral head level through the midpoint and to the S1 root level. **b** Gender differences in the FA values. Blue boxes in men and red boxes in women. **c** Laterality of the FA values. Yellow boxes in the right side and green boxes the left side. **d** A scatterplot of age versus FA values from the femoral nerve, showing an age-dependent decrease ([femoral nerve] = 0.633–0.003 × [age]; *R* ^2^ = 0.092, *p* = 0.03). FH level femoral head level. *p < 0.05

The mean FA values of the normal sciatic nerve were increased distally from the S1 root level, through the midpoint, and to the femoral head level (0.314, 0.446, 0.567, *p* = 0.001, respectively, Fig. 4a). The mean FA values of the femoral nerve (0.565) were similar to the values of the sciatic nerve at the femoral head level. There were no significant differences between men and women for the FA values of the normal sciatic and femoral nerves at any location (Fig. 4b). In addition, there were no significant differences for the FA values with respect to laterality (Fig. 4c). A simple regression analysis revealed an age-dependent decrease in the FA values of the femoral nerve (Fig. 4d).

**Fig. 5:**
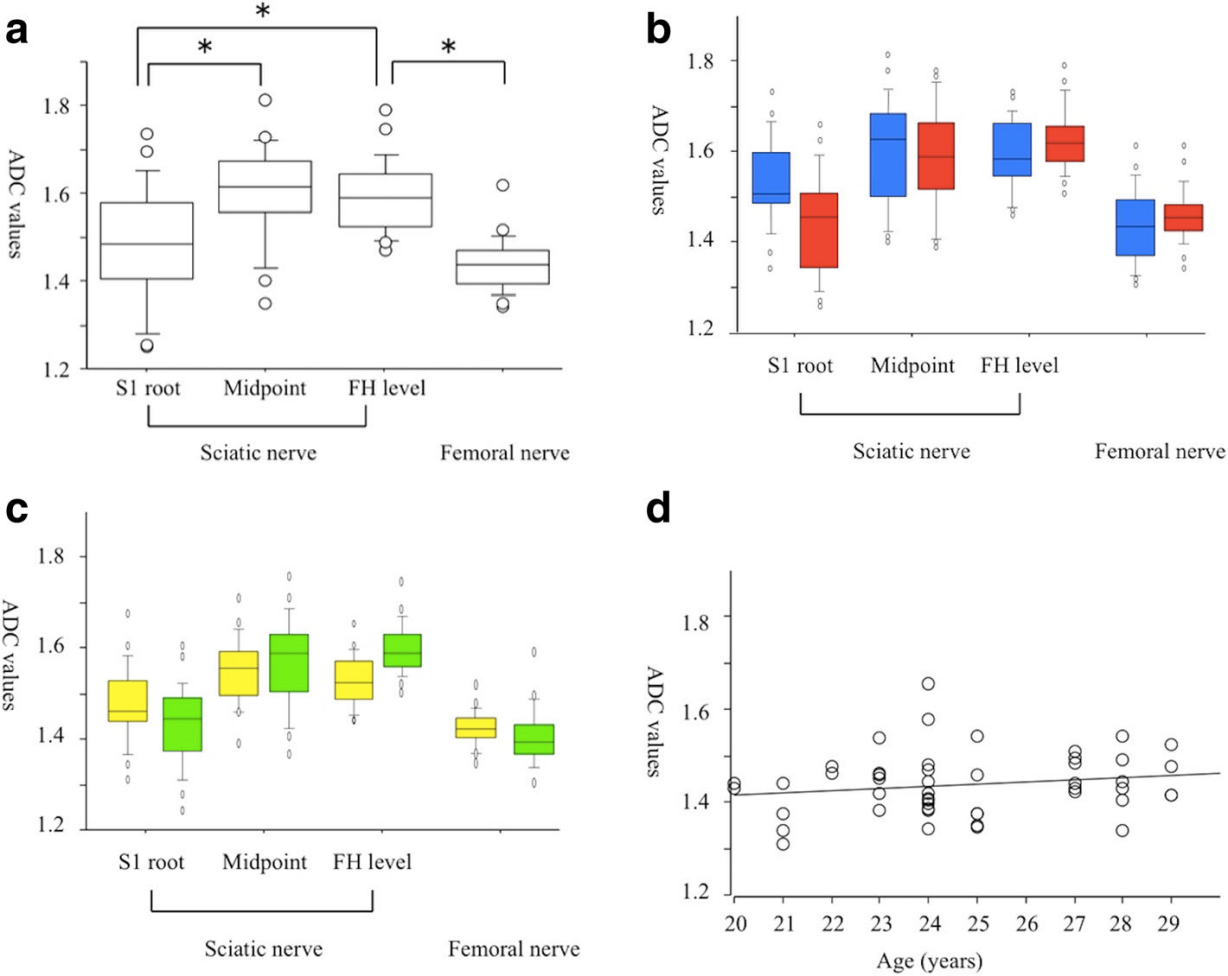
The apparent diffusion coefficient values of the sciatic and femoral nerves. **a** The overall distribution of ADC values. The ADC value of the sciatic nerve was decreased significantly at the S1 root level compared to the femoral head and the midpoint. The femoral nerve also shows low values. **b** Gender differences in the ADC values. Blue boxes in men and red boxes in women. **c** Laterality of the ADC values. Yellow boxes in the right side and green boxes the left side. **d** A scatterplot of age versus ADC values from the femoral nerve, showing no significant correlation. FH level femoral head level. *p < 0.05

The mean ADC values of the normal sciatic nerves were significantly lower in the S1 root level than in the midpoint and the femoral head level (1.481, 1.602, 1.591 × 10^−3^ mm^2^/s, *p* = 0.001, respectively, Fig. 5a). The mean ADC values of the femoral nerve (1.439 × 10^−3^ mm^2^/s) were lower than the values of the sciatic nerve at the femoral head level. There were no significant differences between men and women in the ADC values of the normal sciatic and femoral nerves at any location (Fig. 5b). There were no significant differences in the laterality of the FA values (Fig. 5c) and no relationships between age and ADC values were indicated (Fig. 5d).

## Discussion

To our knowledge, this is the first study to visualize the sciatic and femoral nerves simultaneously around the hip joint with DTI using 3 Tesla MRI. Normal values for FA and ADC in men and women in their twenties were documented. The inter- and intra-observer reliability was considered to be moderate to substantial.

DTI can assess peripheral nerves non-invasively as well as those in the central nervous system. In a preliminary study in 2004, Skorpil et al. [5] successfully visualized the sciatic nerves with DTI on a 1.5-TMR scanner, although the FA and ADC values were not calculated. Kobakci et al. [20] performed the visualization and quantification of the median nerves in 20 healthy volunteers with a calculated FA value of 0.709 and an ADC value of 1.016 × 10^−3^ mm^2^/s. Kobakci et al. [20] also reported that the FA values in patients with carpal tunnel syndrome were less than the normal values by two standard deviations. Kim et al. [6] investigated the sural nerves in 25 healthy volunteers and found the FA values ranged from 0.559 to 0.659. Simon et al. [7] reported an average FA value of the tibial nerve to be 0.45 and that of the peroneal nerve to be 0.43.

In animals, several authors have reported the visualization of the injured sciatic nerve with DTI and that the FA values were decreased significantly after injury but recovered over time [21–23]. In humans, there are few reports describing visualization of the sciatic nerve with DTI. Mathys et al. [1] investigated the utility of DTI for detecting neuropathic changes in sciatic nerve segments of patients with peripheral neuropathy. The mean FA value was significantly lower in patients than in controls (0.433 versus 0.561). They defined the ROI of the sciatic nerve at 10–20 cm cranially from the upper edge of the patella. The FA value of the S1 root was 0.314 in our study and was similar to that indicated in Oikawa’s report (0.33) [10]. Additionally, we found that the FA value increased with more distal locations. Miyagi et al. also reported FA values increased more distally on lumbar nerve roots [24]. On the other hand, there are no previous reports visualizing the femoral nerve with DTI. The mean FA value of the femoral nerve was 0.565 at 3–4 cm proximal from the femoral head level. This value seems reasonable because the femoral nerve is another major nerve of the lower extremities similar to the sciatic nerve. We also found no significant differences in gender or laterality with high reproducibility. Therefore, we suggest that DTI is a reliable imaging method for the sciatic and femoral nerves.

Clinical applications for DTI are of great interest. Potential uses include trauma, demyelinating diseases, radicular pain originating from a lumbar lesion, piriformis syndrome, and so on. DTI is also useful for tumors of the nerve tissue such as brachial plexus tumor [25] or sciatic perineuroma [26]. In particular, we expect to apply DTI for pain research in diagnosis or outcome measure of hip diseases such as osteoarthritis [16, 17] or osteonecrosis [18, 19]. Hip pain is most often localized to the groin (89%), buttock (38%), greater trochanter (27%), anterior thigh (33%), knee (29%), and the lower back (17%) corresponding to the L1 to L5 dermatomes, but referred pain occurs in 55% of cases [27]. In an animal model, dorsal root ganglion (DRG) neurons innervating the hip were distributed on multiple levels (L1–L4) [28]. In a synovitis rat model, calcitonin gene-related peptide immunoreactivity was increased in the DRG [29]. Birnbaum et al. [30] described that the hip is innervated by the obturator, femoral, sciatic, and superior gluteal nerves. Therefore, in patients with hip disease, changes of the sciatic and the femoral nerves might contribute to the diversity of hip pain as a referred pain.

There were several limitations in this study. First, for a technical feasibility study, normal men and women in only their 20s were included. Normal values of other age groups such as the 30s, 40s, and 50s were unknown. Second, sample size was small. However, Julious proposed a sample size of 12 per group rule of thumb for a pilot study [31]. Then, 24 healthy volunteers (12 men and 12 women) in a young age group from 20 to 29 years were enrolled to compare differences in laterality and gender, to reduce age-related effects, and to enhance the quality of the research with cost-effectiveness. Third, DTI in hip region was controversial about signal-to-noise ratio, optimal number of diffusion gradient directions, and spatial variation of diffusion measurements. It is true that our study has a smaller direction number than previous DTI studies of peripheral nerve. However, we followed Eguchi’s previous research with 11 directions [14] and showed high reproducibility and image quality. Thus, we regarded the direction number of 11 as acceptable for a thick nerve like the sciatic nerve and the femoral nerve. Fourth, clinical usefulness of DTI in patients with hip pathology is still unclear. To date, there are no qualitative or quantitative imaging techniques for hip pain. Therefore, the authors have noticed DTI as a biomarker to clarify the pain mechanism. Currently, we are going to compare the normal side and the affected side in patients with osteoarthritis of the head and osteonecrosis of the femoral head. As a technical feasibility study, this study showed DTI of the sciatic and the femoral nerves in healthy volunteers.

## Conclusion

In conclusion, we achieved visualization and quantification of the sciatic and femoral nerves in healthy men and women in their twenties with DTI on a 3 T MRI with excellent inter- and intra-observer reliability. Further research is necessary to compare DTI in the normal and the affected side in patients with hip disorder.

## Abbreviations

ADC: Apparent diffusion coefficient
DTI: Diffusion tensor imaging
FA: Fractional anisotropy
MR: Magnetic resonance
